# Charge to the Graduates of Jefferson Medical College of Philadelphia, Delivered at the Public Commencement, Held March 9th, 1850

**Published:** 1850-04

**Authors:** 


*Charge to the Graduates of Jefferson Medical College of Philadelphia, delivered at the public commencement, held March 9th,* 1850. By J. K. Mitchell, M. D. Published by the Graduating Class.

The Charge of Professor Mitchell, gives an interesting history of the early establishment of Medical schools in Philadelphia, as far back as the year 1765, the first being the medical department of the "College Academy and Charitable School of Philadelphia." It details the founding of the University of Pennsylvania in 1779, in which the old school was merged; of their separation again by legislative enactment in 1789, and of their final union in 1791 under the title of the "Medical Faculty of the University of Pennsylvania." The History of Jefferson Medical College from its foundation in 1824 till the present date, is also briefly given, together with some valuable statistics, from which we learn that,

"For the last seven years, the whole number of matriculants has been 3,184, making an average annual class of 454, whilst the annual average class of the oldest and most distinguished school of our country is 455. Our average class for *six* years, is even higher than that, being 474; whilst the highest average for the same time, in any other school, is 461. It is gratifying to us to perceive from these statistics, that our success has not retarded the progress of our eminent rival, whose average class is steadily on the increase, never having been so high as it is now, in any other six consecutive years of its brilliant existence."

At this particular juncture, when shoals of graduates are emerging from their chrysalis state, it may not be amiss to show by figures, that the danger of being overwhelmed with doctors, which seems to be so much dreaded by the community, is not so imminent as they suppose. By comparing the statistics of Professor

Mitchell with those of Professor Tucker, in this number, the views of both, and the close approximation in the calculation of two careful statisticians, cannot fail to strike the reader.

"The number of medical men in the city and suburbs of Philadelphia is four hundred and ninety-seven; which, supposing the population to be three hundred and fifty thousand, gives one physician for seven hundred and four persons—a proportion about equal to that of the capital of Prussia. If we suppose that the same proportion extends to the country at large, there should be, in a population of twenty-two millions, thirty-one thousand two hundred and fifty physicians. This result is singularly confirmed by the fact that a great publishing house in this city distributes a gratuitous medical monthly paper to upwards of thirty thousand physicians, of whom it has the names and addresses. If each of these physicians continued to practise until he died, and if none of them abandoned the profession, from indolence or the temptations of more lucrative occupations, and if professional exposure and unhealthy places did not exalt the proportional mortality beyond that of the most salubrious residences, four hundred and thirty-nine physicians would die annually in the United States. If we suppose that old age, bad health, the seductions of other employments, and the acquirement of a competency, may carry out of the profession not more than two individuals of every one hundred, or two per cent., the profession will, from all these various causes, lose six hundred and twenty-five persons annually. The increase in the population of the United States by birth and immigration, amounts now, to not less than seven hundred thousand souls annually; for whom, according to the rate assumed, there will be required not less than nine hundred and ninety-four doctors. Thus then, to supply the loss by death, by desertion, and by the annual increase of population, there should be created every year, two thousand and fifty-eight graduates. But the army and navy are to be supplied with physicians, and there must be a large migration of medical men into the newly-acquired territories of the nation. Adventurous physicians are also scattered over the world. One of my private pupils is practising medicine in China, another at Manilla, and a third in California, while two of them are seeking for knowledge in the capital of France.

A great number, perhaps a tenth, of the existing practitioners of the United States, who are among the enumerated 31,250 doctors, are, by ignorance, totally unfit for the duties which they have assumed. They have never seen a college, and many of them have scarcely entered a school of any kind. To supersede such men would demand the creation of at least three thousand graduates in medicine. To say, therefore, that twenty-five hundred physicians should be annually created would be to make an assertion much within the bounds of truth.

A reference to the statistics of the medical schools of the United States, made by an able committee, to the National Medical Association, in May last, shows that the mean number of graduates for the last five years, was twelve hundred and eighty-three, the greatest number being, in any one year, fourteen hundred and twenty-one, and the least, one thousand and thirty-one.

Thus you perceive that scarcely half as many persons receive a degree in medicine, as the wants of the country demand, and that the growth of empiricism is unhappily on the increase, because the expenses of a medical education place its proper attainment beyond the reach of most of the practitioners of the country, or because the masses are not yet sufficiently educated to perceive the priceless value to the community, of a well-instructed physician."

We cordially commend this document to our readers, as it contains much valuable information, and return our thanks to the Professor for the pleasure we have found in its perusal.

